# Genotype-phenotype correlation in neurofibromatosis type-1: *NF1* whole gene deletions lead to high tumor-burden and increased tumor-growth

**DOI:** 10.1371/journal.pgen.1009517

**Published:** 2021-05-05

**Authors:** Lennart Well, Kimberly Döbel, Lan Kluwe, Peter Bannas, Said Farschtschi, Gerhard Adam, Victor-Felix Mautner, Johannes Salamon

**Affiliations:** 1 Department of Diagnostic and Interventional Radiology and Nuclear Medicine, University Medical Center Hamburg-Eppendorf, Hamburg, Germany; 2 Department of Neurology, University Medical Center Hamburg-Eppendorf, Hamburg, Germany; 3 Department of Maxillofacial Surgery, University Medical Center Hamburg-Eppendorf, Hamburg, Germany; University of Miami, Miller School of Medicine, UNITED STATES

## Abstract

Neurofibromatosis type-1 (NF1) patients suffer from cutaneous and subcutaneous neurofibromas (CNF) and large plexiform neurofibromas (PNF). Whole gene deletions of the *NF1* gene can cause a more severe phenotype compared to smaller intragenic changes. Two distinct groups of *NF1* whole gene deletions are type-1 deletions and atypical deletions. Our aim was to assess volumes and averaged annual growth-rates of CNF and PNF in patients with *NF1* whole gene deletions and to compare these with NF1 patients without large deletions of the *NF1* gene.

We retrospectively evaluated 140 whole-body MR examinations of 38 patients with *NF1* whole gene deletions (type-1 group: n = 27/atypical group n = 11) and an age- and sex matched collective of 38 NF1-patients. Age-dependent subgroups were created (0–18 vs >18 years). Sixty-four patients received follow-up MRI examinations (*NF1*whole gene deletion n = 32/control group n = 32). Whole-body tumor-volumes were semi-automatically assessed (MedX, V3.42). Tumor volumes and averaged annual growth-rates were compared.

Median tumor-burden was significantly higher in the type-1 group (418ml; IQR 77 – 950ml, p = 0.012) but not in the atypical group (356ml;IQR 140–1190ml, p = 0.099) when compared to the controls (49ml; IQR 11–691ml). Averaged annual growth rates were significantly higher in both the type-1 group (14%/year; IQR 45–36%/year, p = 0.004) and atypical group (11%/year; IQR 5–23%/year, p = 0.014) compared to the controls (4%/year; IQR1–8%/year). Averaged annual growth rates were significantly higher in pediatric patients with type-1 deletions (21%/year) compared with adult patients (8%/year, p = 0.014) and also compared with pediatric patients without large deletions of the *NF1* gene (3.3%/year, p = 0.0015).

*NF1* whole gene deletions cause a more severe phenotype of NF1 with higher tumor burden and higher growth-rates compared to NF1 patients without large deletions of the *NF1* gene. In particular, pediatric patients with type-1 deletions display a pronounced tumor growth.

## Author summary

Neurofibromatosis type-1 (NF1) is an autosomal dominantly inherited tumor predisposition syndrome and is caused by a variety of mutations of the *NF1* gene. Large deletions encompassing the entire *NF1* gene are known to be associated with a more severe clinical phenotype. A hallmark feature of NF1 is the development of different benign tumors: cutaneous or subcutaneous neurofibromas (CNF) and the potentially large plexiform neurofibromas (PNF). Importantly, PNF can undergo transformation into malignant peripheral nerve sheath tumors, which contribute to the increased mortality of NF1 patients. Identified risk factors for malignant transformation are *NF1* whole gene deletions, large whole-body tumor burden and a high number of subcutaneous neurofibromas. We performed semi-automated volumetry of these tumors to determine volumes and growth rates of CNF and PNF in patients with whole gene deletions and a control group of NF1 patients without large deletions of the *NF1* gene. Our results indicate that *NF1* whole gene deletions lead to higher tumor burden and higher growth rates. In particular, pediatric NF1 patients with a specific type of whole gene deletion (type-1 deletion) exhibit rapid growth of PNF. We therefore suggest that these patients should be monitored in close intervals to evaluate tumor growth and risk of malignant transformation.

## Introduction

Neurofibromatosis type-1 (NF1) is an autosomal dominantly inherited tumor predisposition syndrome with an incidence of 1 in 3000 [1, 2]. NF1 is caused by heterozygous inactivation of the *NF1* tumor suppressor gene on 17q11.2 [3]. A variety of mutations of the *NF1* gene can lead to a manifestation of the disease [4]. Large deletions encompassing the entire *NF1* gene and its flanking regions (*NF1* whole gene deletions) have been identified in 5–11% of patients [5–8].

The most frequent *NF1* whole gene deletions are the so called type-1 deletions accounting for 70–80% of large *NF1* deletions [9, 10]. Type-1 deletions have a size of 1.4Mb and include 14 protein-coding genes as well as four microRNA genes [9–11]. These deletions usually occur as germline mutations, which are present in all cells of affected patients [12, 13]. Less frequently identified are so-called atypical deletions, distinguishable by size, breakpoint location, number of affected genes and frequency of mosaicism [4].

*NF1* whole gene deletions of the *NF1* gene are associated with a more severe clinical phenotype of NF1, including dysmorphic facial features, intellectual disability, congenital cardiac anomalies and increased numbers of cutaneous and subcutaneous neurofibromas (CNF) and plexiform neurofibromas (PNF) [14–17].

The development of CNF and PNF is a hallmark feature of NF1 [18]. PNF can grow to a large size and can cause clinical complications such as skeletal deformation, compression of organs and vessels or neurological impairment [19]. Furthermore, PNF can undergo transformation into malignant peripheral nerve sheath tumors (MPNST) in 8–13% of NF1 patients. *NF1* whole gene deletions have been identified as a risk factor for development of MPNST [20, 21]. Other risk factors for growth of NF1-associated tumors are young age, large whole-body tumor burden, female sex and a high number of subcutaneous neurofibromas [22–24]. To quantify growth of tumors and to assess the potential risk of malignant transformation of PNF to MPNST, NF1 patients repeatedly undergo whole-body MRI examinations [25, 26]. Semi-automated volumetry has been demonstrated to be a reliable tool for quantification of volume and growth of internal tumors [27–30]. However, quantification of CNF by semi-automated volumetry has not been reported as of yet.

Previous studies have shown that *NF1* whole gene deletions can be associated with a higher tumor burden in affected patients compared with patients without large deletions [28]. However, growth rates of tumors in these patients have not been determined.

Therefore, we aimed to assess volumes and averaged annual growth rates of CNF and PNF in NF1 patients with *NF1* whole gene deletions, and to compare these with NF1 patients without large deletions of the *NF1* gene.

## Material and methods

### Ethics statement

This retrospective, Health Insurance Portability and Accountability Act (HIPAA)-compliant study has been approved by the ethical-review board of the Ärztekammer Hamburg (no. PV7214) and complied with the local data protection guidelines as well as the Declaration of Helsinki and its later amendments. Written informed consent was obtained from all participants or their legal representatives.

### Study population

In this study, 38 patients with *NF1* whole gene deletions (mean age 25.8 years; range 4–58 years; 20 females) were included. This group consisted of 27 patients with type-1 deletions (type-1 group; n = 27; mean age 24.6; range 4–53 years; 13 females) and 11 patients with atypical deletions (atypical group; n = 11; mean age 28.7; range 4–58 years; 6 females). These patients (type-1 and atypical) were compared to an age- and sex-matched group of 38 patients without large deletions of the *NF1* gene (control group) (mean age 25.2; range 4–53 years; 20 females). Several patients of this study population (15/76 patients) have been investigated in a previous study (type-1 group: 7/27; atypical group 2/11 patients; control group: 6/38 patients)[28].

Inclusion criteria for this study for both, study group and control group, were fulfillment of the National Institutes of Health (NIH) diagnostic criteria for NF-1 [31], genetic analysis of the *NF1* gene and availability of at least one whole-body MRI examination for volumetry. Exclusion criteria were the inability to undergo MRI examinations or lack of genetic analysis. Additionally, patients with suspected or confirmed mosaic deletions were excluded from this study to prevent a potential bias as genetic mosaicism can induce mild phenotypes. The *NF1* gene mutations for each patient are provided in supporting information **S1** and **S2 Tables**.

All patients were screened for whole gene deletions using multiple intragenic microsatellite markers or directly with a multiple-ligation-dependent primer amplification assay [7, 32] Patients without whole gene deletions were further screened for intragenic minor NF1 mutations by direct Sanger sequencing (Supporting information **S1** and **S2 Tables**) [33]. All patients included received clinical care and genetic counseling at our outpatient clinic.

For subgroup analysis, patients were further divided by age at the timepoint of the first MRI examination into children/adolescents ≤18 years of age (type-1 group: n = 8; mean age 11.0 years, range 4–18 years / atypical group: n = 3; mean age 9.3 years; range 4–15 years / control group: n = 12; mean age 11.1 years; range 3–18 years) and adults > 18 years of age (type-1 group: n = 19; mean age 30.3 years; range 19–53 years / atypical group: n = 8; mean age 36 years; range 21–58 years / control group: n = 26; mean age 31.7 years; range 19–56 years)[29].

### MRI examinations and volumetry

A total of 140 MRI examinations of all included patients were performed as part of the standard clinical care between September 2003 and March 2019 at 1.5 T or 3 T (Siemens Avanto or Skyra, Siemens Healthineers, Erlangen, Germany). MRI-sequences were T1w TSE coronal (1.5 T:TR 572 ms; TE 9 ms; FA 90°; in plane resolution 0.88 x 0.88; slice thickness 6 mm / 3 T: TR 731 ms; TE 11 ms; FA 160°; in plane resolution 0.98 mm x 0.89 mm; slice thickness 7 mm), T2w TIRM coronal (1.5 T: TR 3110 ms; TE 101 ms; FA 150°; in plane resolution 0.94 mm x 2.1 mm; slice thickness 7 mm / 3 T: TR 4000 ms; TE 45 ms; FA 130°; in plane resolution 1.29 mm x 1.03 mm; slice thickness 7 mm), T2w HASTE TIRM axial (1.5 T: TR 4910 ms; TE 104 ms; FA 150°; in plane resolution 0.96 mm x 1.9 mm; slice thickness: 8 mm / 3 T: TR 1200 ms; TE 85 ms, FA 148°; in plane resolution 1.17 mm x 1.17 mm; slice thickness 8 mm) and T2w TSE sagittal (1.5 T: TR 4180 ms; TE 102 ms; FA 170°; in plane resolution 0.63 mm x 1.25 mm; slice thickness 3.5 mm /3 T: TR 4600 ms; TE 96 ms; FA 160°; in plane resolution 0.68 mm x 0.63 mm; slice thickness 3 mm) sequences. No contrast enhanced sequences were performed.

32/38 patients (84.2%) of the study group (type-1 group: n = 22/27; atypical group: n = 10/11) and 32/38 patients (84.2%) of the control group received follow-up MRI examinations with a mean time interval between examinations of 6.3 years in the study group (range 2.0–12.7 years) and 5.5 years in the control group (range 2.0–9.8 years). 12 patients received only one MRI examination (type-1 group: 5/27; atypical group: 1/11; control group: 6/32).

### Image analysis and volumetry

All MRI examinations were evaluated regarding the presence of CNF or PNF including rare discrete internal tumors (smaller singular nodular lesions)[22]. PNF and discrete internal tumors represent the patient’s internal tumor load[22].

**Fig 1:**
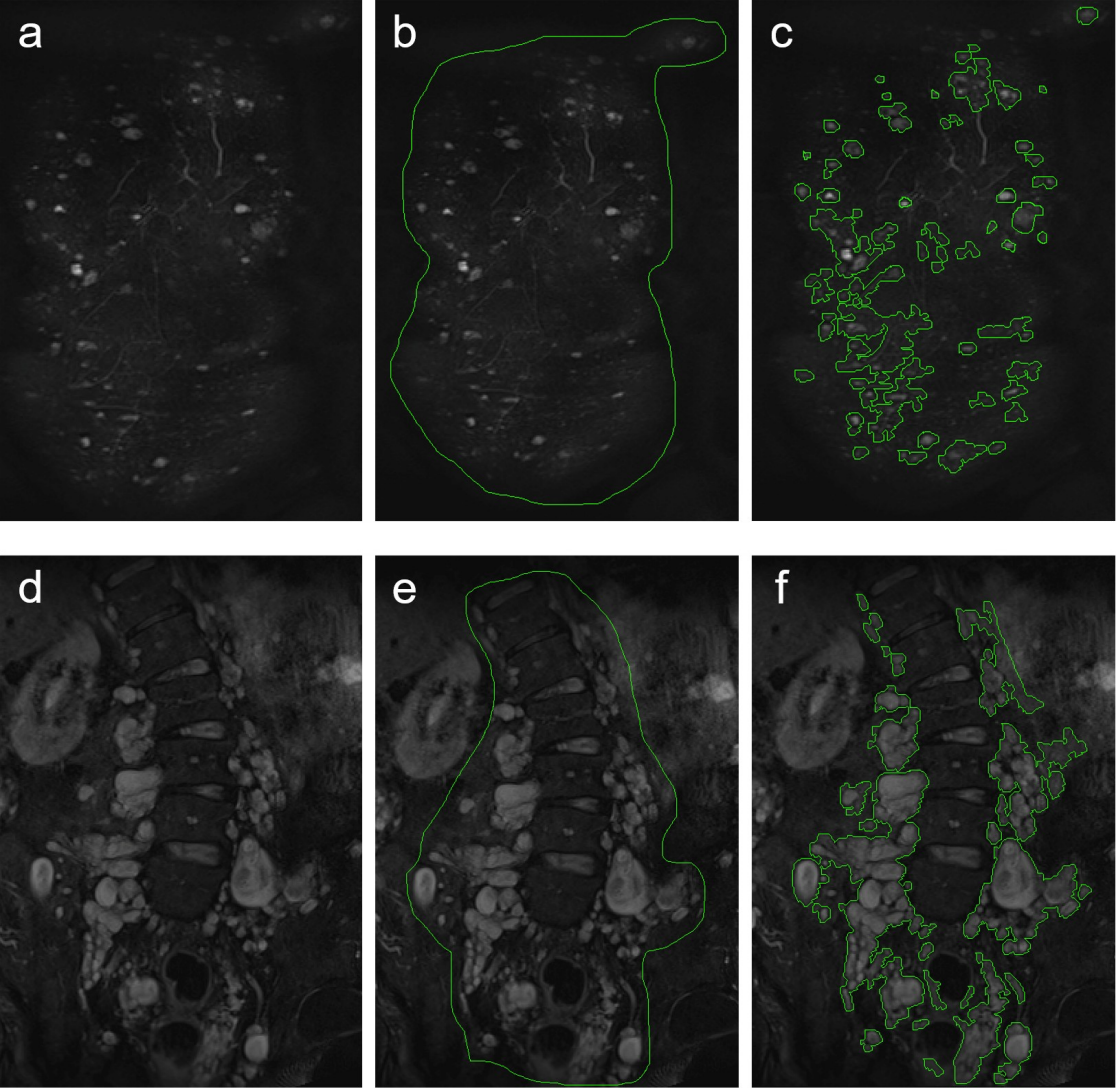
**Illustration of semi-quantitative volumetry of cutaneous and subcutaneous neurofibromas (CNF) (a-c) and plexiform neurofibromas (PNF) (d-f).** Displayed are fat-saturated T2-weighted coronal MR images of a 28-year-old female patient with a high number of CNF **(a-c)** and of a 32-year-old male patient with large formations of paraspinal plexiform neurofibromas **(d-f)**. Readers identified CNF **(a)** and PNF **(d)** according to their characteristic morphology. Readers then manually placed a region of interest, encompassing the identified tumors **(b, e)**. The software then automatically contoured individual lesions **(c, f)**.

CNF were identified as singular circular-shaped and homogenous hyperintense lesions on T2-weighted images with random cutaneous or subcutaneous distribution [34, 35] (**Fig 1A–1C**). It was not possible to satisfactorily distinguish cutaneous neurofibromas from subcutaneous ones, therefore the two entities were measured as one group. PNF were identified by their characteristic appearance as polylobulated, hyperintense masses on T2-weighted images spreading along peripheral nerves [29, 34] (**Fig 1D and 1E**).

Volumetry of tumors was performed using MedX software (V3.42) on fat-saturated T2-weighted images (**Fig 1**). MedX utilizes a heuristic-based semi-automated method for segmentation and measurement [27]. Previous studies have restricted volumetry of tumors to tumor sizes of ≥ 3 cm [29]. To evaluate if volumetry of smaller tumors is feasible and reproducible with MedX, a set of gelatin phantoms was built: Disposable syringes were filled with dissolved gelatin (Rainin Pipet-Lite XLS) (volumes: 0.1ml, 0.2ml, 0.3ml, 0.4ml, 0.5ml, 1.0ml, 2ml) and stored at 4°C overnight. The syringes were then cut open and the hardened gelatin was extracted (Supporting information **S1 Fig**). MRI scans of these phantoms were performed, using the coronal T2-weighted TIRM sequence described above (Supporting information **S3 Table**). Volumes were then measured for three times by one reader as described below (Supporting information **S3 Table**). The measured volumes did not significantly differ from the known volume of the phantoms (p = 0.74).

For volumetry of patients, tumors were identified in the coronal plane according to their above-mentioned morphology (**Fig 1A and 1D**). In a second step, a region of interest was manually drawn encompassing the identified tumors (**Fig 1B and 1E**). The software then semi-automatically contoured individual lesions (**Fig 1D and 1F**). If semi-automated measurement was not feasible, tumors were traced manually using the built-in drawing tool of the MedX software[27]. The volumetry process is illustrated in **Fig 1**. Volumetry was performed separately for CNF and PNF in each patient which enabled determination of separate volumes for each. Summation of CNF and PNF volumes enabled calculation of whole-body tumor volume. Ten patients (5 type-1 group / 5 control group) were randomly selected and volumetry was repeated by a second reader to evaluate inter-rater reliability. Individual growth rates were calculated as the averaged annual growth in all patients with available follow-up examinations [24].

### Statistical analysis

Continuous variables derived from tumor volumetry are presented as median ± interquartile range (IQR). All variables were evaluated for normal distribution by the Shapiro-Wilk test. Tumor volumes (ml) were compared between the groups using the Mann-Whitney U rank sum test due to not normal distribution. Prevalence of tumors in both groups was compared using Fisher’s exact test. Measured volumes of tumor phantoms were compared with the actual volume by a paired Student’s t-test. The intraclass correlation coefficient and coefficient of variation were calculated from repeated volumetry of patients. All tests were two-sided. Growth of tumors was calculated if two MRI examinations were available. Growth rate is given as averaged annual growth in % per year. An increase in tumor volume > 20% per year was considered as progressive tumor growth [24, 29]. Tumor growth rates were compared using the Mann-Whitney U rank sum test due to not normal distribution. Patients who did not display tumors or subsets of tumors were excluded from calculation of tumor volumes and growth rates. Statistical analysis was carried out using GraphPad Prism 5.0 for Windows. P values < 0.05 were considered significant.

## Results

### Prevalence and distribution of tumors

Prevalence of CNF and PNF was significantly higher in the *NF1* whole gene deletion group (CNF: 37/38 patients; PNF: 36/38 patients) than in the control group (CNF: 27/38 patients; PNF: 28/38 patients)(both p < 0.0001).

There was no statistically significant difference between the prevalence of CNF in the type-1 group (26/27 patients), the atypical group (11/11 patients) and the control group (27/38 patients) (all p > 0.05).

There was also no statistically significant difference between the prevalence of PNF in the type-1 group (26/27 patients), the atypical group (10/11 patients) and the control group (28/38 patients) (all p > 0.05). The prevalence of CNF and PNF within the age-dependent subgroups is provided in **Table 1**.

**Table 1:** Prevalence of cutaneous and subcutaneous neurofibromas or plexiform neurofibromas in age-dependent subgroups of patients with either type-1 deletions of the *NF1* gene, atypical deletions or without large deletions of the *NF1* gene (control).

tumor type	patient group	type-1 deletion	atypical deletion	control
**cutaneous and subcutaneous** **neurofibromas**	children/ adolescents	7/8	3/3	6/12
	adults	19/19	8/8	21/26
**plexiform** **neurofibromas**	children/ adolescents	8/8	2/3	9/12
	adults	18/19	8/8	19/26

Presented are numbers of patients in the respective subgroups.

Malignant peripheral nerve sheath tumors were detected in three of the 38 patients with *NF1* whole gene deletions within the observed time period (type-1 group: n = 2; atypical group: n = 1), resulting in a prevalence of 7.9% (type-1 group: 7.4%; atypical group 9.1%), respectively. None of the patients of the control group developed malignant peripheral nerve sheath tumors.

### Tumor volumes

**Fig 2:**
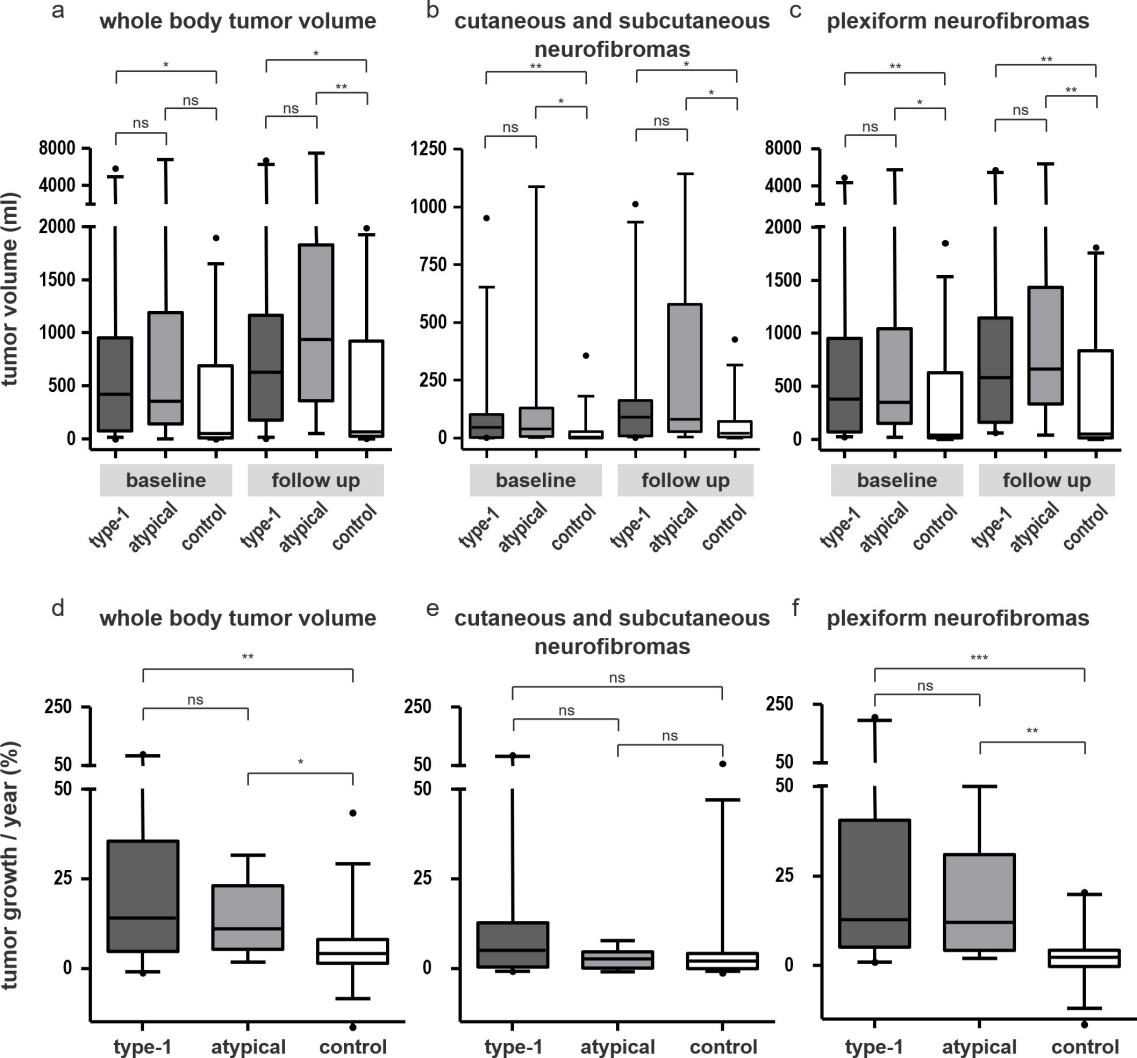
**Comparison of tumor volumes (a-c) and averaged annual growth-rates of tumors (d-f) in NF1 patients with type-1 deletions, atypical deletions and patients without large deletions of the *NF1* gene (control) at baseline and at follow-up.** Displayed are volumes of whole-body tumor burden **(a)**, cutaneous and subcutaneous neurofibromas **(b)** and plexiform neurofibromas **(c)** and averaged annual growth rates of whole-body tumor volume **(d)**, cutaneous and subcutaneous neurofibromas (CNF) **(d)** and plexiform neurofibromas (PNF) **(f)**.

Median whole-body tumor volumes at baseline examination were: 418 ml (IQR 77–950 ml) in the type-1 group, 356 ml (IQR 140–1190 ml) in the atypical group and 49 ml (IQR 11–691 ml) in the control group. Volumes were significantly higher in the type-1 group than in the control group (p = 0.012) (**Fig 2A**). No significant differences were found between the type-1 group and the atypical group (p = 0.87) or between the atypical group and the control group (p = 0.099).

Volumes of CNF at baseline were: 46 ml (IQR 1–102 ml) in the type-1 group, 40 ml (IQR 6–129 ml) in the atypical group and 6 ml (IQR 0.1–26 ml) in the control group. Volumes of CNF were significantly higher in the type-1 group than in the control group (p = 0.007) and higher in the atypical group than in the control group (p = 0.013) (**Fig 2B**).

Volumes of PNF were: 382 ml (IQR 69–952 ml) in the type-1 group, 348 ml (IQR 153–1043 ml) in the atypical group and 41 ml (14–630 ml) in the control group. Volumes of PNF were significantly higher in the *NF1* whole gene deletion groups than in the control group at baseline and at follow-up (**Fig 2C**) (all p<0.05). This difference was not significant when comparing the type-1 group and the atypical group (p = 0.79).

Outliers of large tumor volumes > 3000 ml were identified in the type-1 group (n = 2) and in the atypical group (n = 1) but not in the control group (type-1 group vs control group: p = 0.17; atypical group vs control group: p = 0.22).

Subgroup analysis revealed a significantly higher whole-body tumor volume in the type-1 group compared with the control group in adult patients (p = 0.019). There was no significant difference between these groups in children/adolescents (p = 0.07) at baseline (**Table 2**). Volumes of CNF were significantly higher in the type-1 group than in the control group in adult patients at baseline (p = 0.003) and at follow-up (p = 0.037) whereas volumes of CNF did not significantly differ between the groups of children / adolescents (all p > 0.05) (**Table 2**). Also, volumes of PNF were significantly higher in the type-1 group than in the control group in both children / adolescents and adult patients at baseline (all p < 0.05) (**Table 2**).

**Table 2:** Comparison of median tumor volumes at baseline and at follow-up in age-dependent subgroups of NF1 patients with type-1 deletions vs NF1 patients without large deletions of the *NF1* gene (control).

**baseline**	**tumor type**	**patient group**	**n**	**type-1 deletion (ml)**	**n**	**control (ml)**	**p value**
	whole-body tumor volume	children/ adolescents	8/8	**170.8** (55.2–782.1)	12/12	**33.2** (1.0–226.9)	*0*.*07*
		adults	19/19	**492.7** (192.4–1312)	26/26	**58.8** (10.7–805.8)	*0*.*019*
		p value		*0*.*13*		*0*.*26*	
	cutaneous and subcutaneous neurofibromas	children/ adolescents	7/8	**1.3** (1.0–76.3)	6/12	**0.2** (0–10.2)	*0*.*25*
		adults	19/19	**66.5** (36.9–116.4)	21/26	**8.1** (0.8–32.7)	*0*.*003*
		p value		*0*.*047*		*0*.*12*	
	plexiform neurofibromas	children/ adolescents	8/8	**106.9** (37.5–780.9)	9/12	**25.3** (4.1–49.1)	*0*.*041*
		adults	18/19	**471** (166.8–1526)	19/26	**131** (17.6–805.4)	*0*.*044*
		p value		*0*.*07*		*0*.*06*	
**follow-up**	whole-body tumor volume	children/ adolescents	8/8	**389** (195.7–1962)	11/11	**58.0** (24.3–399.3)	*0*.*046*
		adults	14/14	725.8 (135.4–1167)	21/21	101.9 (18.0–1019)	*0*.*062*
		p		*0*.*811*		*1*.*0*	
	cutaneous and subcutaneous neurofibromas	children/ adolescents	7/8	**62.6** (7.1–174.2)	6/11	**4.2** (1.0–67.2)	*0*.*10*
		adults	14/14	94.1 (26.5–162.1)	21/21	24.5 (7.8–71.2)	*0*.*037*
		p		*0*.*61*		*0*.*16*	
	plexiform neurofibromas	children/ adolescents	8/8	**285.1** (129.2–1576)	9/11	**46.5** (2.1–127.6)	*0*.*006*
		adults	14/14	**625.1** (173.2–1145)	19/21	**129.2** (18.7–989.5)	*0*.*099*
		p		*0*.*54*		*0*.*18*	

Values in parentheses represent interquartile range.

Details of all tumor volumes of age-dependent subgroups at baseline and at follow- up are provided in **Table 2**. Volumetric results for each patient are provided in supporting information **S4 Table**. Results of the repeated volumetry for evaluation of inter-rater reliability are provided in S5 Table.

### Growth dynamics

Median averaged annual growth rates for whole-body tumor burden were: 14%/year (IQR 4.8–35.5) in the type-1 group, 11%/year (IQR 5.3–22.9) in the atypical group and 4.2%/year (IQR 1.4–8.1) in the control group. The averaged annual growth rates of whole-body tumor burden in the type-1 group and the atypical group were not significantly different (p = 0.67), but were significantly higher than in the control group (p = 0.004 and p = 0.014) (**Fig 2D**).

Averaged annual growth rates of CNF were: 5.1%/year (IQR 0.4–12.) in the type-1 group, 2.5%/year (IQR 0.1–4.6) in the atypical group and 2.1%/year (IQR 0–4.1) in the control group. There were no statistically significant differences between the three groups (all p > 0.05) (**Fig 2E**).

Averaged annual growth rates of PNF were: 12.7%/year (IQR 5.1–40.4) in the type-1 group, 12.1%/year (IQR 4.2–31) in the atypical group and 2.2%/year (IQR -0.3–4.3) in the control group. Averaged annual growth rates of PNF were not significantly different between the *NF1* whole gene deletion groups (p = 0.75) but were significantly higher than in the control group (type-1 group vs control group: p < 0.0001 / atypical group vs control group: p < 0.0016) (**Fig 2F**).

### Progressive tumor growth

Progressive tumor growth (> 20%/year) of whole-body tumor volume and of PNF was detected in 8/22 patients in the type-1 group, in 3/10 patients in the atypical group and in 2/32 patients in the control group.

The prevalence of progressive tumor growth was significantly higher in the type-1 group than in the control group (p = 0.01). The prevalence was not significantly different between the atypical group and the control group (p = 0.08) or between the type-1 group and the atypical group (p = 1.0).

**Fig 3:**
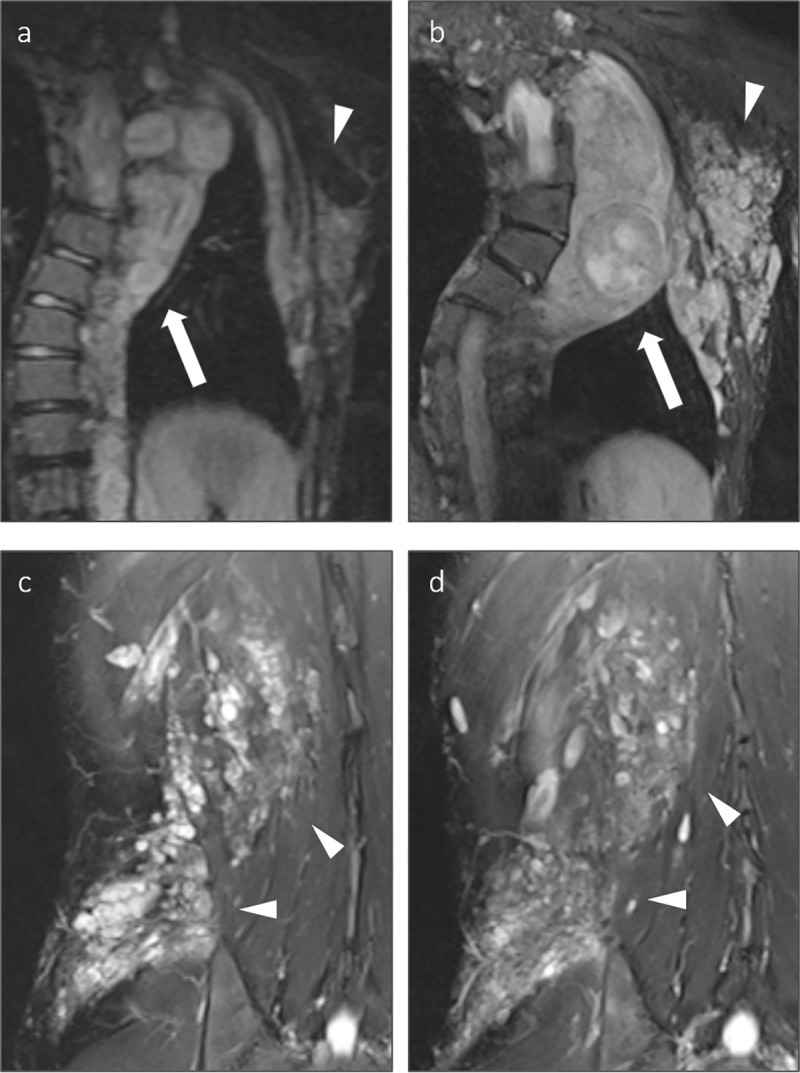
**Exemplary presentation of tumor growth in a 12-year-old, type-1 deletion patient (a and b) and a 19-year-old patient with an intragenic mutation of the *NF1* gene (c and d).** Displayed are coronal T2-weighted, fat saturated MR images of the thorax (a, b) and the right flank (c, d). The type-1 deletion patient displayed significant growth of a paraspinal plexiform neurofibroma of the thorax with paraspinal and axillary extension, including a distinct nodular lesion within the plexiform neurofibroma (arrow). The interval between the examinations was eight years. In contrast, the patient with an intragenic mutation displayed no significant growth of the plexiform neurofibroma of the right flank (arrowheads) over an interval of five years.

Progressive tumor growth (> 20%/year) of CNF was detected in 3/22 patients in the type-1 group and in 2/32 patients in the control group whereas no patient in the atypical group showed progressive growth of CNF (all p > 0.05). An exemplary illustration of progressive tumor growth is provided in **Fig 3**.

### Subgroup analysis of tumor growth

For subgroup analysis of tumor growth, only patients of the type-1 group and the control group were compared, due to the small number of patients in the subgroup of atypical children / adolescents with follow-up MRI examinations (n = 2). Subgroup analysis of patients with type-1 mutations included n = 8 children/adolescents and n = 14 adults. Subgroup analysis of patients without large deletions included n = 11 children/adolescents and n = 21 adults.

Highest growth rates of whole-body tumor volume were detected in the subgroup of type-1 children / adolescents with 21%/year (IQR 14.8–47.6) (**Table 3**). These growth rates were significantly higher than those of adult patients in the type-1 group (median growth rate 8%/year; IQR 1.4–19.1) (p = 0.014) and higher than those of children /adolescents from the control group (median growth rate 3.3%/year; IQR 0.6–7.1) (p = 0.015) (**Table 3**). Similarly, growth rates of both CNF (median growth rate 13.9%/year; IQR 3–45) and PNF (19.4%/year; IQR 12.2–78.4) were significantly higher in children / adolescents in the type-1 group than in any of the other subgroups (all p < 0.05) (**Table 3**).

**Table 3:** Median averaged annual growth rates of tumors in age-dependent subgroups of NF1 patients with type-1 deletions vs NF1 patients without large deletions of the *NF1* gene (control).

tumor type	patient group	n	type-1 deletion	n	control	p
whole-body tumor averaged annual growth rate (%)	children/ adolescents	8/8	**21.0** (14.8–47.6)	11/11	**3.3** (0.6–7.1)	*0*.*0015*
	adults	14/14	**8.0** (1.4–19.1)	21/21	**4.3** (2.0–8.5)	*0*.*31*
	p		*0*.*014*		*0*.*43*	
averaged annual growth rate of cutaneous and subcutaneous neurofibromas (%)	children/ adolescents	7/8	**13.9** (3.0–45)	6/11	**0** (0–4.1)	*0*.*047*
	adults	14/14	**1.8** (0.1–6.9)	21/21	**2.2** (0–4.1)	*0*.*69*
	p		*0*.*03*		*0*.*90*	
averaged annual growth rate of plexiform neurofibromas (%)	children/ adolescents	8/8	**19.4** (12.2–78.4)	9/11	**0** (-0.6–3.7)	*0*.*0005*
	adults	14/14	**8.3** (2.5–25.5)	19/21	**2.8** (-0.3–5.1)	*0*.*013*
	p		*0*.*041*		*0*.*51*	

Values in parentheses represent interquartile range.

## Discussion

In this study, we demonstrated that NF1 caused by whole gene deletions of the *NF1* gene leads to a more severe phenotype with higher tumor burden and higher averaged annual growth rates when compared with NF1 without large deletions of the *NF1* gene. In particular, pediatric patients with type-1 deletions display a pronounced averaged annual growth of cutaneous and plexiform neurofibromas.

The prevalence of CNF and PNF in our study was high in the *NF1* whole gene deletion group (37/38 and 36/38 patients (97% and 95%)) as well as in the control group (27/38 and 28/38 patients (71%and 74%)) and thus higher than that reported in previous studies (76–85% and 50–76%)[14, 28, 29, 36]. The higher prevalence of tumors in our study population is potentially caused by the comparatively high number of patients who received whole-body MRI scans due to clinical complaints while presenting at our clinic. The prevalence of MPNST in patients with whole-gene deletions in our study (7.9%) was comparable to others, e.g. that reported by Pasmant et al (7.1%). The prevalence of MPNST in patients without large deletions of the *NF1* gene in our study (0%) was lower than that reported by Pasmant et al (3.1.%), however this bias might be caused by the small number of patients included[16].

Whole-body tumor volume as well as volumes of CNF and PNF in our study were significantly higher in patients with type-1 deletions than in the control group. The median tumor volume (387 ml) in our patients with *NF1* whole gene deletions was similar to previously reported whole-body tumor volumes in patients with *NF1* whole gene deletions (321 ml)[28]. Furthermore, the whole-body tumor volume in our control group (49 ml) was similar to the tumor volume of the general NF1 population previously reported (18.7–107.9 ml)[22, 28, 29].

In contrast to our findings, a previous study by Kluwe et al did not identify significant differences in volumes of PNF between NF1 patients with *NF1* whole gene deletions and patients without large deletions of the *NF1* gene [28]. This difference in results is potentially caused by divergent inclusion criteria and divergent volumetric processing between the studies as Kluwe et al included patients with genetic mosaicism and performed volumetry only on tumors that were larger than 3 cm in longest diameter. However, in that study the prevalence of patients with a high tumor burden (> 3000 ml) was significantly increased in patients with *NF1* whole gene deletions [28]. We similarly identified more patients with a high tumor burden (> 3000 ml) in the *NF1* whole gene deletion groups, but this difference was not statistically significant when compared to patients without large deletions of the *NF1* gene.

Subgroup analysis of tumor volumes revealed a significantly higher whole-body tumor volume and higher volumes of CNF in adult patients with type-1 deletions compared with patients in the control group. More importantly, both adults and children / adolescents with type-1 deletions displayed significantly higher volumes of PNF than age- and sex-matched patients without large deletions of the *NF1* gene, further supporting the assertion that type-1 deletions are associated with a more severe phenotype. The observation that young patients with type-1 deletions display higher volumes of PNF than young patients without large deletions not only supports the hypothesis that type-1 deletions of the *NF1* gene are associated with a more severe phenotype of the disease, but also that this effect occurs at a young age [14–17].

We were able to demonstrate that volumetry of CNF is feasible with MedX, which has previously not been reported. Volumes of CNF were significantly higher in patients with large deletions of the *NF1* gene compared with the control group. Subgroup analysis only identified a significantly higher volume of CNF in adult patients with type-1 deletions and not in children / adolescents, which contrasts the subgroup analysis of PNF. This finding underlines the different biological characteristics of PNF and CNF, as PNF are embryonically developed tumors with a different growth pattern[37].

Averaged annual growth rates of tumors were significantly higher in patients with *NF1* whole gene deletions in our study. This was true for averaged annual whole-body tumor-growth and averaged annual growth of PNF, but not for averaged annual growth of CNF. Most importantly, children / adolescents with type-1 deletions showed significantly higher averaged annual growth rates of both CNF and PNF when compared with their age- and sex-matched controls or with adult patients with type-1 deletions. In comparison, patients without large deletions of the *NF1* gene did not display such an accentuated averaged annual tumor-growth at a young age. The averaged annual growth rates of PNF in patients with type-1 deletions in our study (14%) were similar to the reported growth rates of PNF by Dombi et al (14.3%)[24] and by Akshintala et al. (13.9%)[38]. These studies did not provide genotype information of the included patients. However, due to the inclusion of children and young adults with substantial tumor burden seeking treatment on clinical trials, the similar growth rates seem plausible and do not necessarily represent the average growth rates in the entirety of NF1 patients[24, 38].

Additionally, more patients in the type-1 deletion group (8/22 patients) and in the atypical deletion group (3/10 patients) displayed progressive tumor growth of PNF of > 20%/year compared with the control group (2/32 patients). The overall prevalence of progressive tumor growth in the *NF1* whole gene deletion group of 34.4% (13/32 patients) was higher than that reported for the general NF1 population by Nguyen et al (13.5%)[29]. Patients without large deletions of the *NF1* gene displayed lower average annual growth rates in our study, similar to the general NF1 population investigated by Nguyen et al[29]. These observations further emphasize the importance of the underlying genetic profile when investigating tumor burden and growth in NF1.

Our subgroup analysis showing an increased averaged annual tumor-growth in children / adolescents with type-1 deletions goes beyond previous studies [24, 29, 38, 39] and has important clinical implications: We detected this significantly higher averaged annual tumor growth only in patients with type-1 deletions but not in patients without large deletions of the *NF1* gene. It can be reasoned that the previously reported increase of tumor growth at a young age is mainly caused by the presence of patients with type-1 deletions in the general population [24, 29, 38, 39]. The identified higher tumor burden in patients with type-1 deletions is of special interest with regard to a possible treatment of inoperable or clinically symptomatic PNF with MEK-inhibitors (MEKi) [40]. Our observation of a high prevalence of tumors and of increased averaged annual growth of tumors in young patients with type-1 deletions might additionally encourage clinicians to re-evaluate the current clinical practice. Type-1 deletions, large numbers and volumes of tumors and high growth rates have all been identified as potential risk factors for the development of MPNST [14, 20, 21, 29, 41]. Furthermore, patients with *NF1* whole gene deletions are at an elevated risk of developing MPNST [42]. The findings of this study might therefore strengthen the argument for regular MRI examinations in young patients with type-1 deletions, to monitor tumor growth and potential malignant transformation of tumors. This hypothesis should be evaluated in further prospective studies.

We did not identify statistically significant differences in averaged annual growth of tumors between NF1 patients with type-1 deletions and those with atypical deletions. This might be expected, as atypical deletions often are heterozygous and associated with genetic mosaicism, which results in a variety of phenotypes of NF1, ranging from severe forms, similar to that of the type-1 deletion to mild forms, which are clinically almost indetectable[32]. However, due to the exclusion of patients with known or suspected genetic mosaicism in this study, phenotypical differences between type-1 deletions and atypical deletions might be limited in the investigated cohort.

Since the time between the follow-up MRI examinations was variable, the averaged annual growth rates identified in this study do not necessarily represent the actual growth rates of tumors in each year included in the observed time period. As Akshintala et al. have described, tumor growth rates can vary within patients over time and some plexiform neurofibromas can also demonstrate spontaneous decrease in volume[38]. Larger, long term prospective studies with fixed intervals between follow-up MRI examinations are required to better understand the growth dynamics of NF1 associated tumors in correlation with the genotype of patients.

There are limitations to our study. Due to its retrospective nature, the time interval between baseline and follow-up examinations is not identical in all patients. It would be favorable to examine patients prospectively within a defined time frame with predefined time intervals. However, with the selection of a control group that was not only matched in age and sex but also in time interval between examinations, the effect of different observed time periods may be neglected.

Another limitation is the limited number of patients investigated, resulting in relatively small subgroups. In particular, a larger patient cohort would be favorable for the analysis of atypical *NF1* deletions. Since atypical NF1 deletions occur in only 8–10% of large *NF1* deletions, larger patient groups are difficult to obtain [4]. Additionally, our volumetry approach did not allow a sufficient distinction between cutaneous and subcutaneous neurofibromas as both tumor entities appear as hyperintense nodules in proximity to the skin. Our approach also does not provide total numbers of cutaneous and subcutaneous neurofibromas and can therefore not differentiate between growth of preexisting tumors and new appearance of these lesions. Despite this weakness, we demonstrate that MRI-based volumetry is a useful method to assess the total volumes of these tumors. It might be of interest to additionally investigate the numbers of cutaneous and subcutaneous neurofibromas in future studies, which was not feasible with the software used here.

In conclusion, NF1 caused by *NF1* whole gene deletions leads to a more severe phenotype with higher tumor burden and higher tumor growth rates when compared to NF1 patients without large deletions of the *NF1* gene. In particular, pediatric NF1 patients with type-1 deletions exhibit rapid growth of disease-associated tumors. We therefore suggest that these patients should be monitored in close intervals to evaluate tumor growth, risk of malignant transformation of tumors and with regard to evaluation for MEKi treatment.

## Supporting information

S1 Table Individual results of the analysis of *NF1* gene mutations in patients with *NF1* whole gene deletions. (XLSX)

S2 Table Individual results of the analysis of NF1 gene mutations in patients without large deletions of the *NF1* gene. (XLSX)

S3 Table Volumetric results of gelatin phantoms with indicated SD. Measurements were performed with MedX (V3.42). Calculated volumes represent means derived from three separate measurements.
(XLSX)

S4 Table Individual volumetric results for all patients. (XLSX)

S5 Table Volumetric results of patients who were analyzed by two readers with the calculated intraclass correlation coefficients for whole body tumor volume, cutaneous neurofibromas and plexiform neurofibromas. (XLSX)

S1 Fig Gelatin phantoms (**a**, left column), T2 weighted MRI scan of phantoms (**a**, right column) and results of three volume measurements with indicated SD (**b**), performed with MedX (v3.42).
(TIF)
