# Supplementary figures and images for: Genotype-phenotype correlation in neurofibromatosis type-1: NF1 whole gene deletions lead to high tumor-burden and increased tumor-growth
Source: PLoS Genet. 2021 May 5;17(5):e1009517. doi: 10.1371/journal.pgen.1009517 (PMC8099117; doi:10.1371/journal.pgen.1009517)

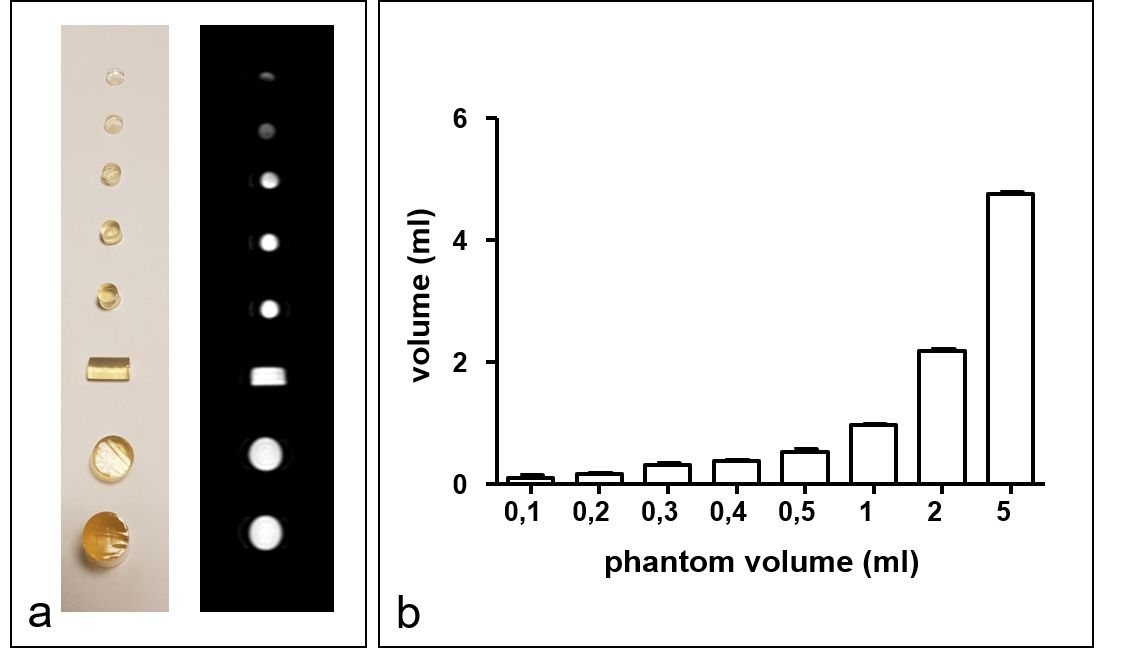

Supplement: S1 Fig — Gelatin phantoms (a, left column), T2 weighted MRI scan of phantoms (a, right column) and results of three volume measurements with indicated SD (b), performed with MedX (v3.42). (TIF) [file pgen.1009517.s006.tif]
